# Giant hiatus hernia in a young person: an unusual case report

**DOI:** 10.1093/jscr/rjag659

**Published:** 2026-10-30

**Authors:** Sima Rajaei

**Affiliations:** Student Research Committee, Faculty of Medicine, Hormozgan University of Medical Sciences, 5th Floor, Bolor Building, Gol Afshan Alley, Moallem St., Madar Blvd., Jomhuri Eslami Blvd., Farhangian Alley, opposite Shahid Mohammadi Hospital, Bandar Abbas, Hormozgan 7916133577, Iran

**Keywords:** hernia, hiatal, stomach diseases, gangrene, gastrectomy, young adult

## Abstract

Giant paraesophageal hernia is an uncommon condition that typically affects older adults and may rarely progress to gastric ischemia or necrosis. A 28-year-old man with no prior comorbidities, trauma, reflux symptoms, or known hiatal hernia is presented. He developed acute intolerance to oral intake, left upper quadrant pain, unproductive retching, dyspnea, fever, tachycardia, and hypoxemia. Imaging demonstrated a giant paraesophageal hernia with 70%–80% of the stomach herniated into the thoracic cavity, without evidence of gastric volvulus. Urgent exploratory laparotomy revealed extensive gastric gangrene extending to the antrum. Total gastrectomy with Roux-en-Y esophagojejunostomy and primary diaphragmatic repair were performed. The postoperative course was favorable, and oral intake was resumed after confirmation of an intact anastomosis. This case demonstrates that life-threatening gastric gangrene can occur in young adults with paraesophageal hernia, even in the absence of volvulus.

## Introduction

Giant paraesophageal hernia is a rare but potentially life-threatening hiatal hernia, involving herniation of a large portion of the stomach, sometimes with other abdominal organs, through the esophageal hiatus into the thoracic cavity [1]. It predominantly occurs in older adults due to age-related connective tissue weakening, diaphragmatic muscle attenuation, and enlargement of the esophageal hiatus [2], making its occurrence in young patients uncommon. Clinical presentation varies from asymptomatic or nonspecific chronic symptoms (epigastric pain, postprandial fullness, nausea, vomiting, dysphagia) to acute complications. Serious outcomes include gastric incarceration, obstruction, volvulus, ischemia, and necrosis [3]. Acute gastric volvulus may present with Borchardt’s triad: severe epigastric pain, unproductive retching, and inability to pass a nasogastric tube [4]. Given the rarity of gastric necrosis, especially in young patients, this case underscores the importance of high clinical suspicion for prompt diagnosis and management.

## Case report

A 28-year-old man with no known medical comorbidities presented to the emergency department with a 2-day history of progressive intolerance to oral intake and left upper quadrant abdominal pain. The pain was deep, aching, non-radiating, and moderate to severe in intensity. He also reported unproductive retching and dyspnea. The patient had no history of upper gastrointestinal symptoms or previous abdominal or thoracic surgery. On arrival (01:17 AM on Day 1), the patient appeared acutely ill. His vital signs showed blood pressure of 105/75 mmHg, heart rate of 120 beats/min, respiratory rate of 22 breaths/min, temperature of 39°C, and oxygen saturation of 92% on room air. Abdominal examination revealed localized tenderness in the left upper quadrant without rebound tenderness or guarding. Chest auscultation demonstrated asymmetric bilateral air entry, while the remainder of the physical examination was unremarkable. Initial laboratory evaluation revealed leukocytosis with a white blood cell count of 12.8 × 10^3^/μl, hemoglobin of 10.5 g/dl, platelet count of 153 × 10^9^/l, random blood glucose of 109 mg/dl, and serum creatinine of 1.10 mg/dl. Serum electrolytes were within normal limits. Venous blood gas analysis demonstrated uncompensated respiratory acidosis, with a pH of 7.27, PaCO₂ of 57 mmHg, and HCO₃^−^ of 25 mmol/l. Chest radiography (03:25 AM on Day 1) showed a left-sided intrathoracic opacity suggestive of a large hiatal hernia (Fig. 1). Computed tomography (CT) (06:51 AM on Day 1). confirmed a huge rolling (paraesophageal) hiatus hernia, with ~70%–80% of the stomach herniated into the thoracic cavity (Fig. 2). A nasogastric tube was successfully inserted for gastric decompression. The patient was resuscitated with intravenous fluids, broad-spectrum antibiotics, and nasogastric decompression. Given the clinical and radiologic concern for a complicated giant paraesophageal hernia with suspected gastric ischemia, the patient’s abdominal pain progressively worsened, accompanied by clinical signs of peritonitis. Considering the urgent need for surgical intervention and the concern that additional endoscopic evaluation would delay definitive treatment without changing management, upper gastrointestinal endoscopy was deferred, and the patient was prepared for emergency exploratory laparotomy (10:00 AM Day 1). Intraoperatively, a 2-cm left diaphragmatic defect adjacent to the esophagus was identified. The stomach was found herniated into the thoracic cavity, with extensive gastric gangrene extending to the antrum. No gastric volvulus, perforation, or gross peritoneal contamination was observed (Fig. 3). Total gastrectomy with Roux-en-Y esophagojejunostomy was performed. The diaphragmatic defect was repaired primarily with polypropylene sutures without mesh reinforcement, and tube thoracostomy was placed before abdominal closure. The postoperative course was favorable with gradual clinical improvement. A contrast study performed on postoperative day 10 confirmed an intact esophagojejunal anastomosis, after which oral intake was initiated. Written informed consent was obtained from the patient for publication of this case.

**Figure 1:**
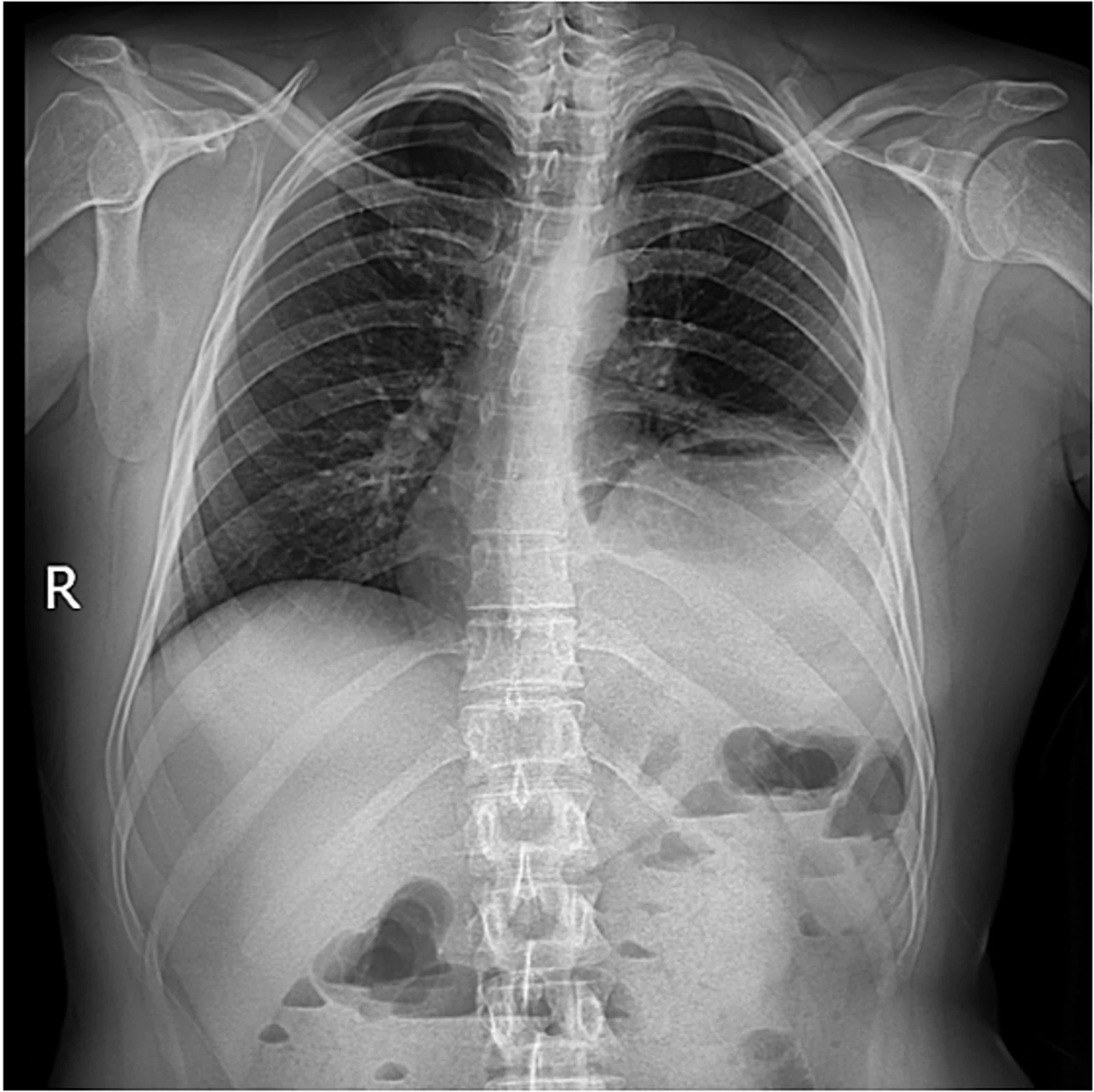
Chest radiograph showing a retrocardiac air–fluid level in the left lower thorax, suggestive of a large hiatal hernia.

**Figure 2:**
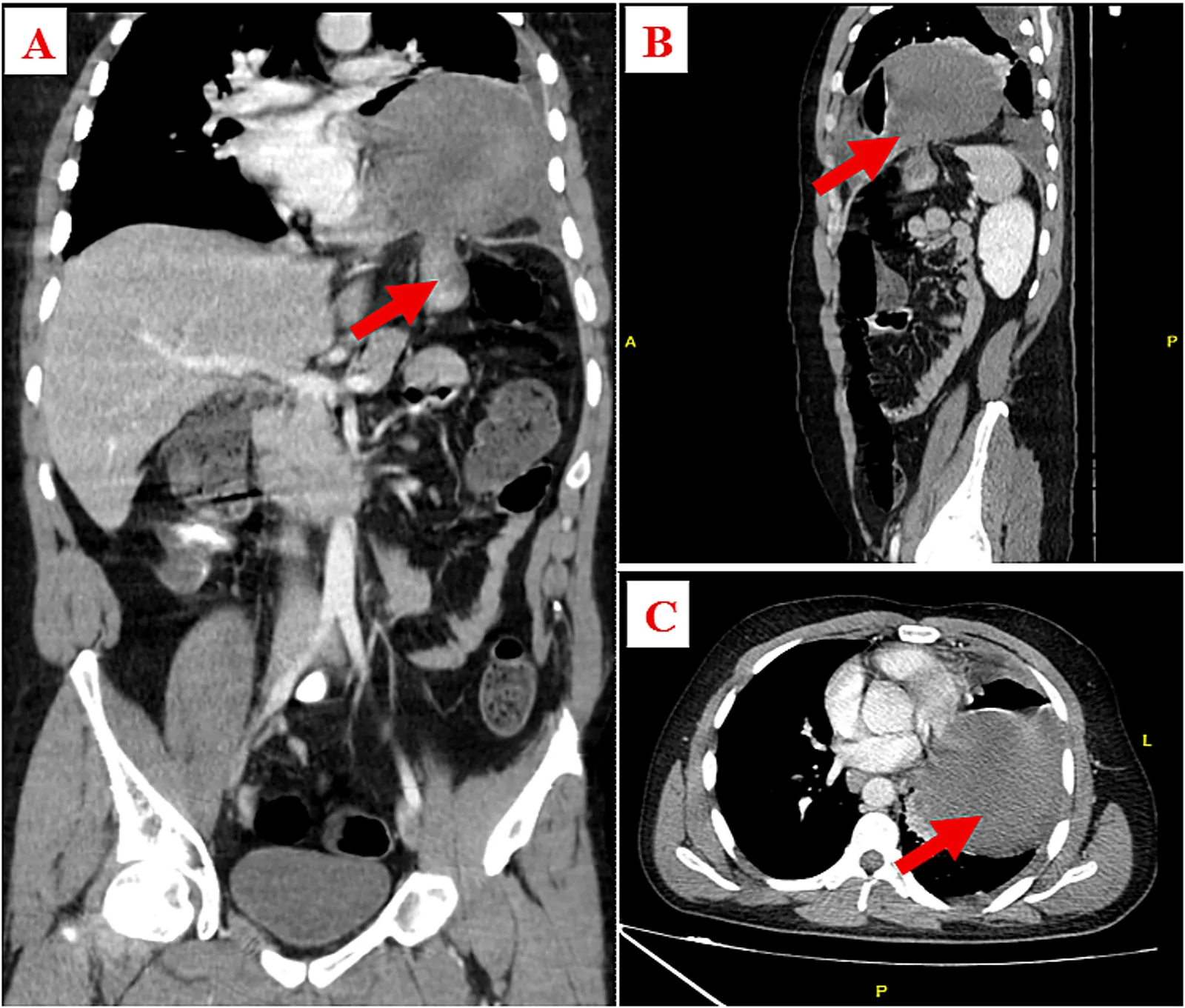
Contrast-enhanced CT of the chest and abdomen demonstrating a large hiatal hernia. (A) Coronal view showing herniation of the stomach through the esophageal hiatus into the thoracic cavity. (B) Sagittal view demonstrating the intrathoracic position of the herniated stomach. (C) Axial view showing the herniated stomach within the thoracic cavity. Arrows indicate the hiatal hernia.

**Figure 3:**
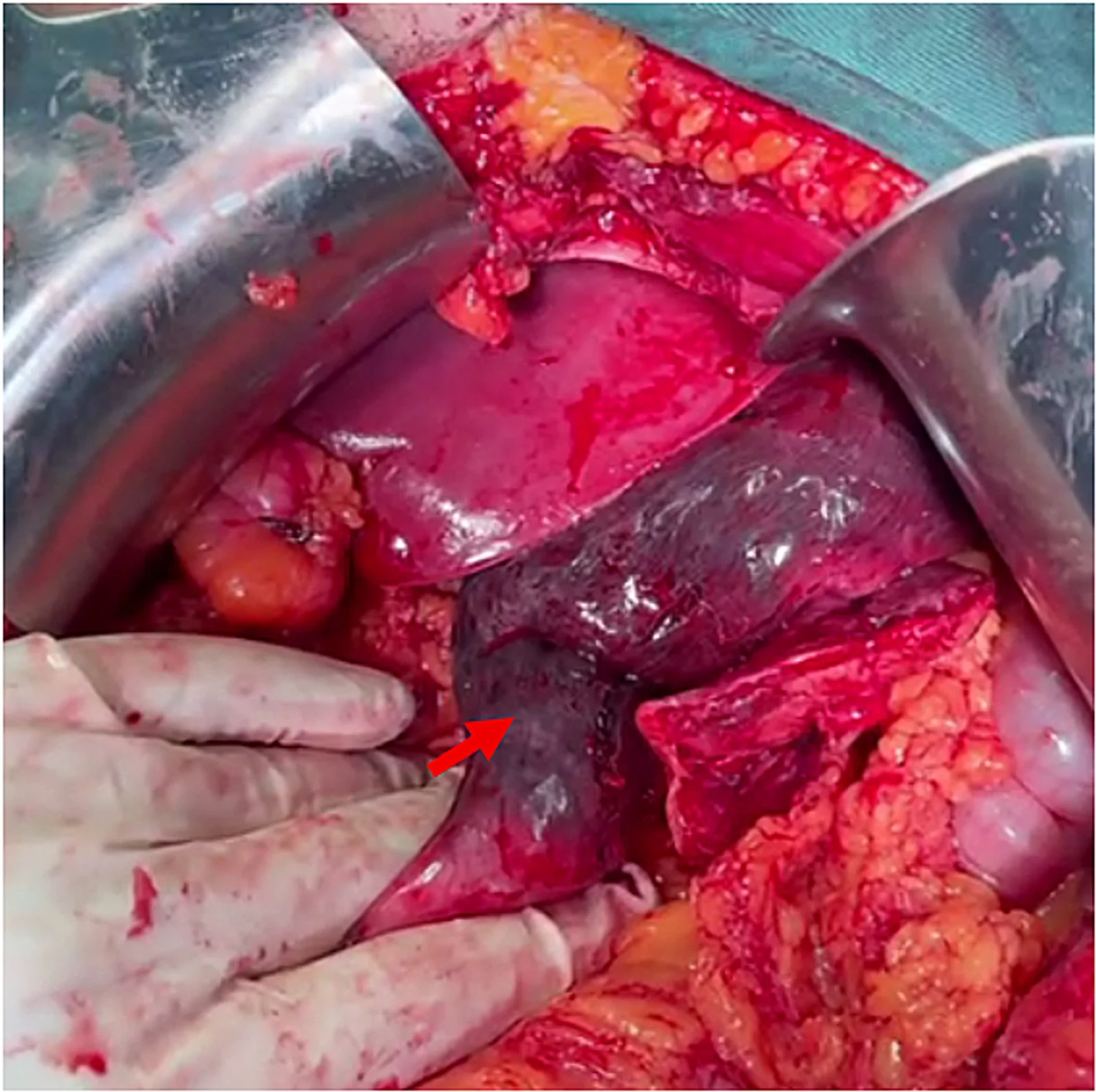
Gross appearance of the stomach (arrow).

## Discussion

This case is clinically remarkable because extensive gastric gangrene occurred in a young adult with a giant type II paraesophageal hernia despite the absence of typical predisposing factors such as trauma, prior thoracoabdominal surgery, reflux symptoms, or a known hiatal hernia. The unusual age and atypical presentation may lower early clinical suspicion, especially when respiratory symptoms and abnormal thoracic imaging predominate.

The most important pathophysiologic feature in this patient was gastric gangrene without radiologic or intraoperative evidence of gastric volvulus. Although necrosis associated with paraesophageal hernia is most often attributed to volvulus, strangulation, or incarceration [5, 6], the operative findings in this case suggest that fixed intrathoracic incarceration alone may be sufficient to cause progressive vascular compromise. Initial impairment of venous outflow can increase intramural pressure, subsequently compromise arterial inflow, and culminate in transmural ischemia and gangrene. This is particularly noteworthy because gastric ischemia is generally uncommon owing to the stomach’s extensive collateral blood supply [7]. Therefore, absence of volvulus should not be considered reassuring when systemic inflammatory signs, feeding intolerance, thoracoabdominal pain, respiratory symptoms, and a large intrathoracic stomach coexist.

Imaging was pivotal in narrowing the diagnosis and expediting operative management. Chest radiography may suggest the diagnosis by showing a retrocardiac or intrathoracic opacity, but CT is more informative in acute settings because it delineates the hernia type, quantifies the intrathoracic stomach, and evaluates complications such as obstruction, ischemia, pneumatosis, perforation, or mediastinal extension [8, 9]. Upper gastrointestinal endoscopy is the primary diagnostic modality for symptomatic hiatal hernias and plays an important role in assessing hernia anatomy, mucosal viability, and associated pathology, as well as in preoperative planning for elective surgical repair. However, in patients with suspected gastric ischemia or strangulation who exhibit progressive clinical deterioration or signs of peritonitis, endoscopy should not delay definitive surgical intervention.

Society of American Gastrointestinal and Endoscopic Surgeons guidelines recommend repair of symptomatic hiatal hernias, particularly in patients with obstruction, reflux, aspiration, or acute complications [10]. In this emergency case, extensive gastric gangrene involving the antrum required total gastrectomy with Roux-en-Y esophagojejunostomy, while primary crural repair without mesh was preferred because of contamination and infection risk.

This case expands the clinical spectrum of complicated paraesophageal hernia by demonstrating that catastrophic gastric ischemia can occur in young adults and even in the absence of volvulus. Its principal limitations are the single-case design and the lack of long-term follow-up data regarding recurrence, nutritional status, and functional outcomes.
